# Supplementary figures and images for: Investigating the effects of radiation, T cell depletion, and bone marrow transplantation on murine gut microbiota
Source: Front Microbiol. 2024 Jun 5;15:1324403. doi: 10.3389/fmicb.2024.1324403 (PMC11188301; doi:10.3389/fmicb.2024.1324403)

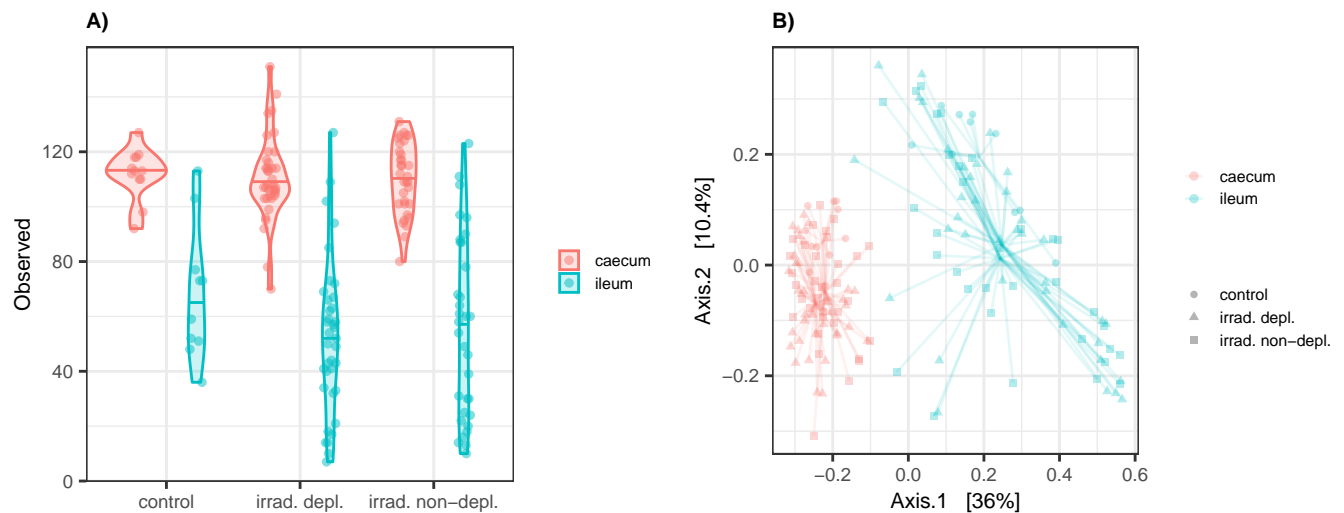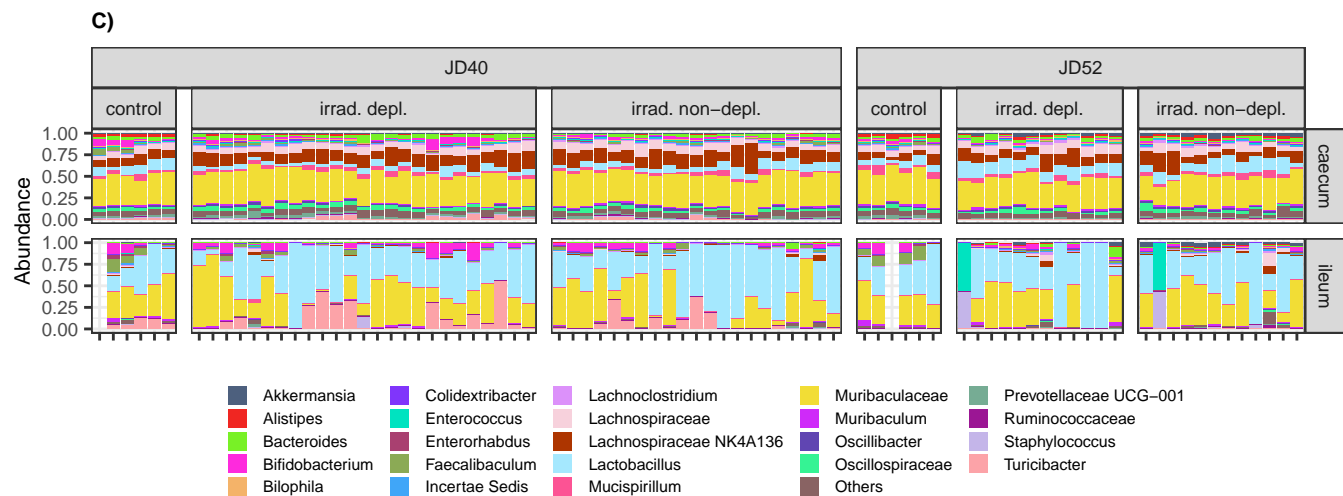

Supplement: SUPPLEMENTARY FIGURE S1 — [Caecum_ileum_overview.pdf]: Differences in gut microbiota between caecum and ileum. Consistently for all experimental groups, the microbiota of the ileum showed a lower alpha diversity and a different composition than the microbiota of the caecum. This is shown by (A) violin plots visualizing the differences in ASV richness, (B) Principal Coordinate analysis for Bray-Curtis dissimilarities, where caecum and ileum samples form non-overlapping clusters and where identity with the experimental group is indicated by Harwestman plots, and (C) bar charts showing the proportions of the 20 most abundant bacterial genera in each sample. [file Data_Sheet_1.PDF]

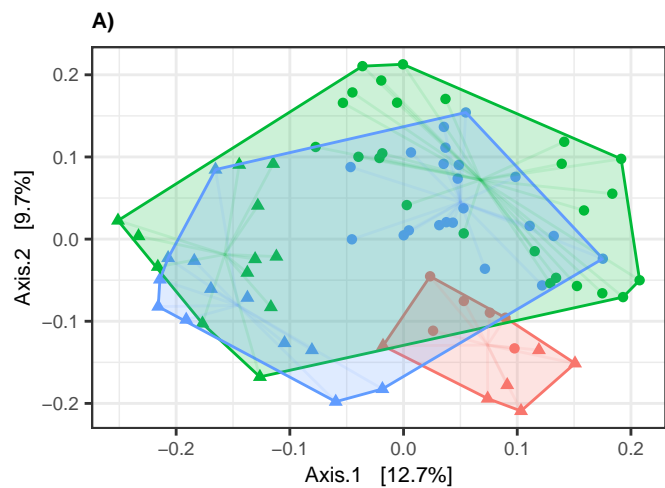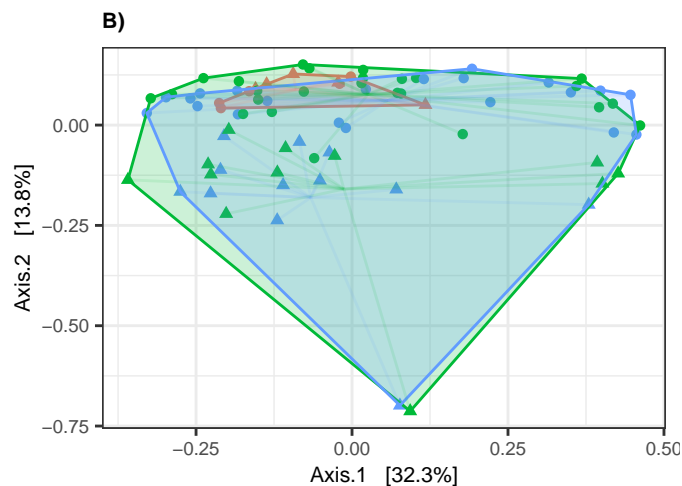

● JD40 ▲ JD52

control depleted non\_depleted

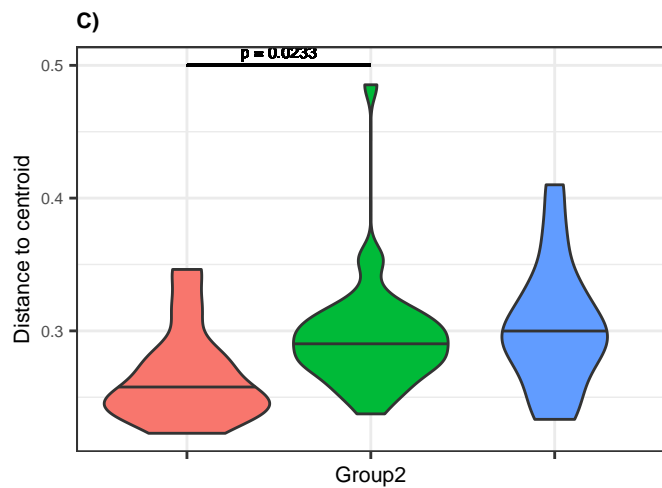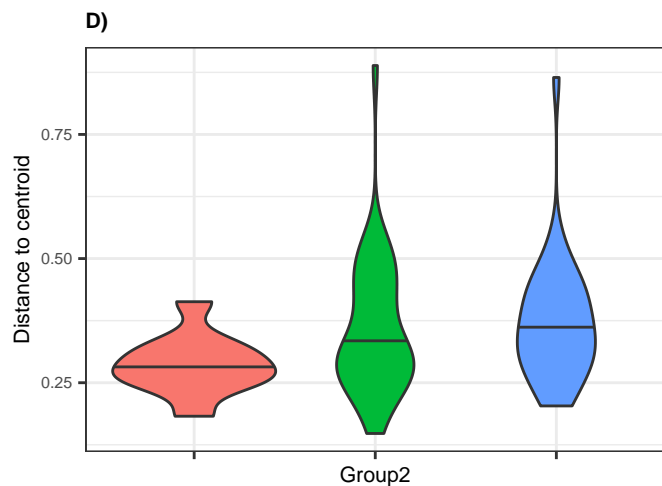

control depleted non\_depleted

Supplement: SUPPLEMENTARY FIGURE S2 — [BETA_all.bc.pdf]: Differences in microbiota composition between non-irradiated controls and depleted or non-depleted mice exposed to radiation. Shifts in the composition of the microbiota of (A) cecum and (B) ileum between treatment groups were analyzed by PCoA ordination of Bray-Curtis dissimilarities. Treatment levels are indicated by different colors. Harwestman diagrams connect samples from the same treatment replicate. Interindividual variation for each treatment group is represented for (C) caecum and (D) ileum samples by violin plots showing the distribution of Bray-Curtis-based distances to the group-specific centroids. The horizontal lines within the violin plots correspond to the median values. Horizontal lines above the violin plots indicate significant differences between the experimental groups. [file Data_Sheet_2.PDF]

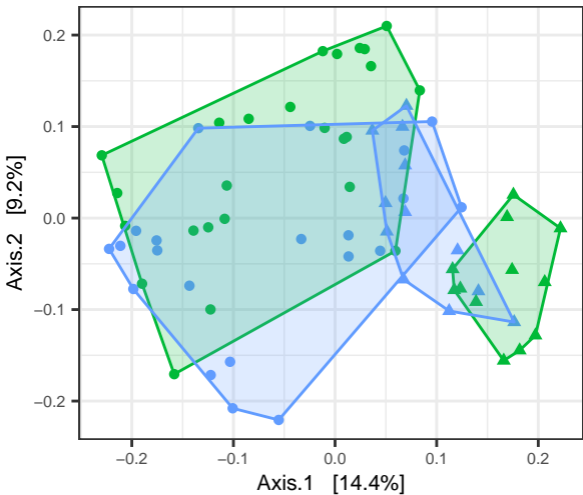

● depleted ● non\_depleted

● high ▲ low

Supplement: SUPPLEMENTARY FIGURE S3 — [Bray_dose.pdf]: PCoA ordination of Bray-Crutis dissimilarities depicts variation in appendix microbiota composition between depleted and non-depleted mice exposed to either high or low doses of radiation. [file Data_Sheet_3.PDF]

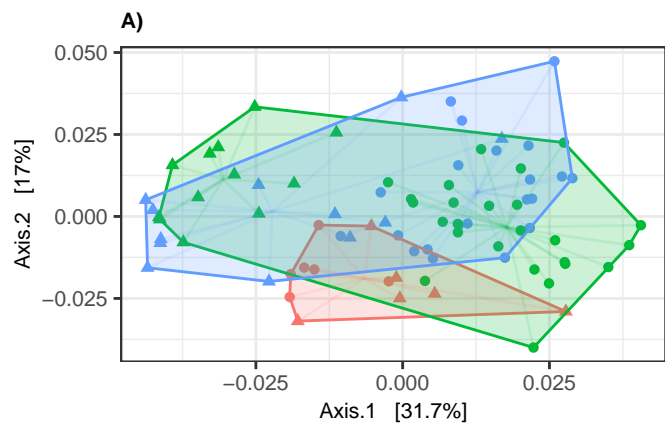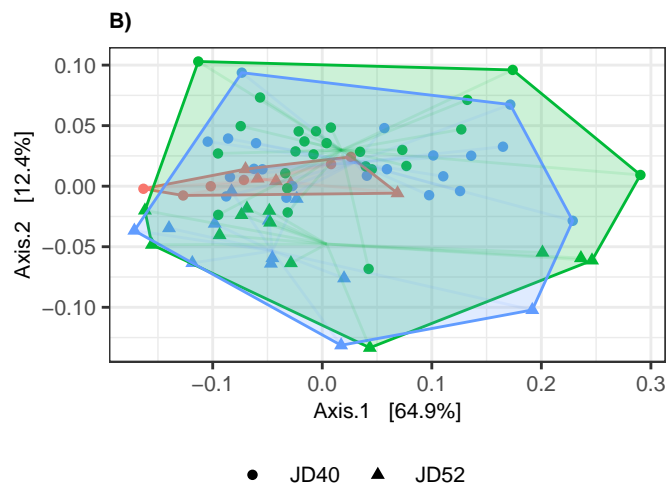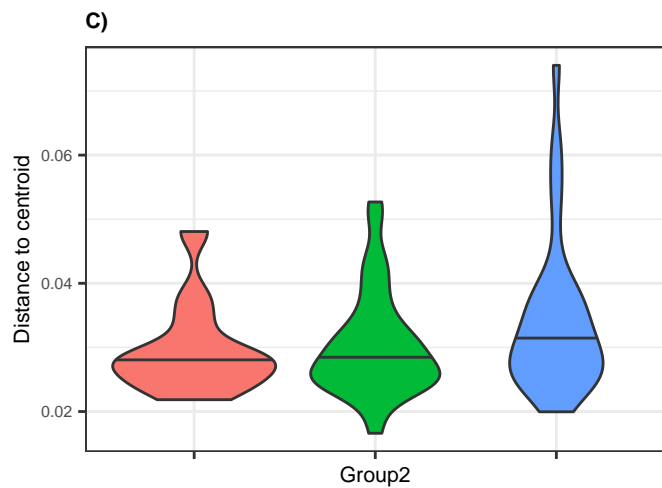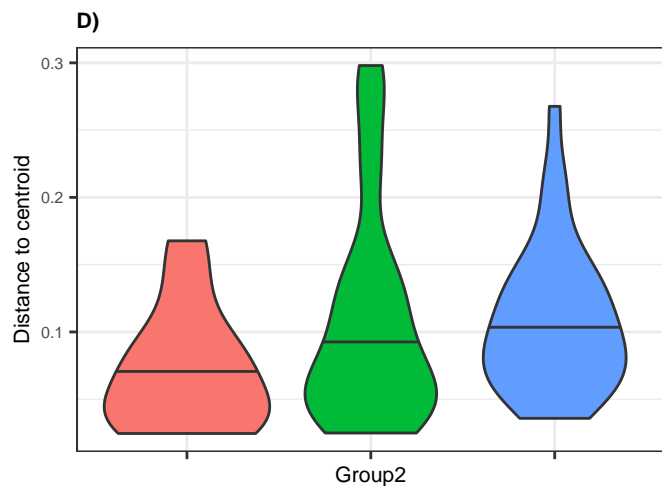

Supplement: SUPPLEMENTARY FIGURE S4 — [BETA_all.bc_METAG.pdf]: Variation in predicted metagenome content of (A) caecum and (B) ileum between treatment groups, assessed based on PCoA for Bray-Curtis dissimilarities. [file Data_Sheet_4.PDF]

Axis.2 [15.8%]

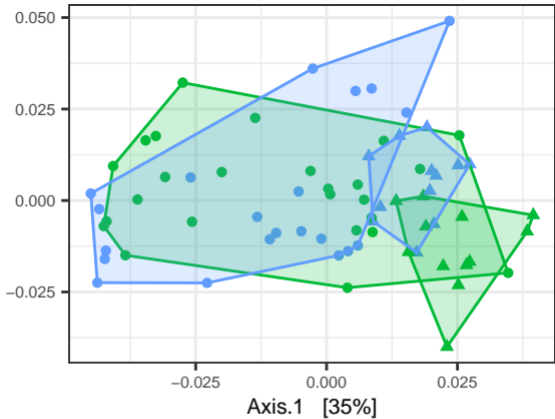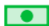

depleted

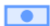

non\_depleted

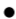

high

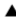

low

Supplement: SUPPLEMENTARY FIGURE S5 — [Bray_dose_METAG.pdf]: PCoA ordination of Bray-Curtis dissimilarities showing the differences in the composition of predicted cecal metagenomes between depleted and non-depleted mice exposed to either high or low doses of radiation. [file Data_Sheet_5.PDF]

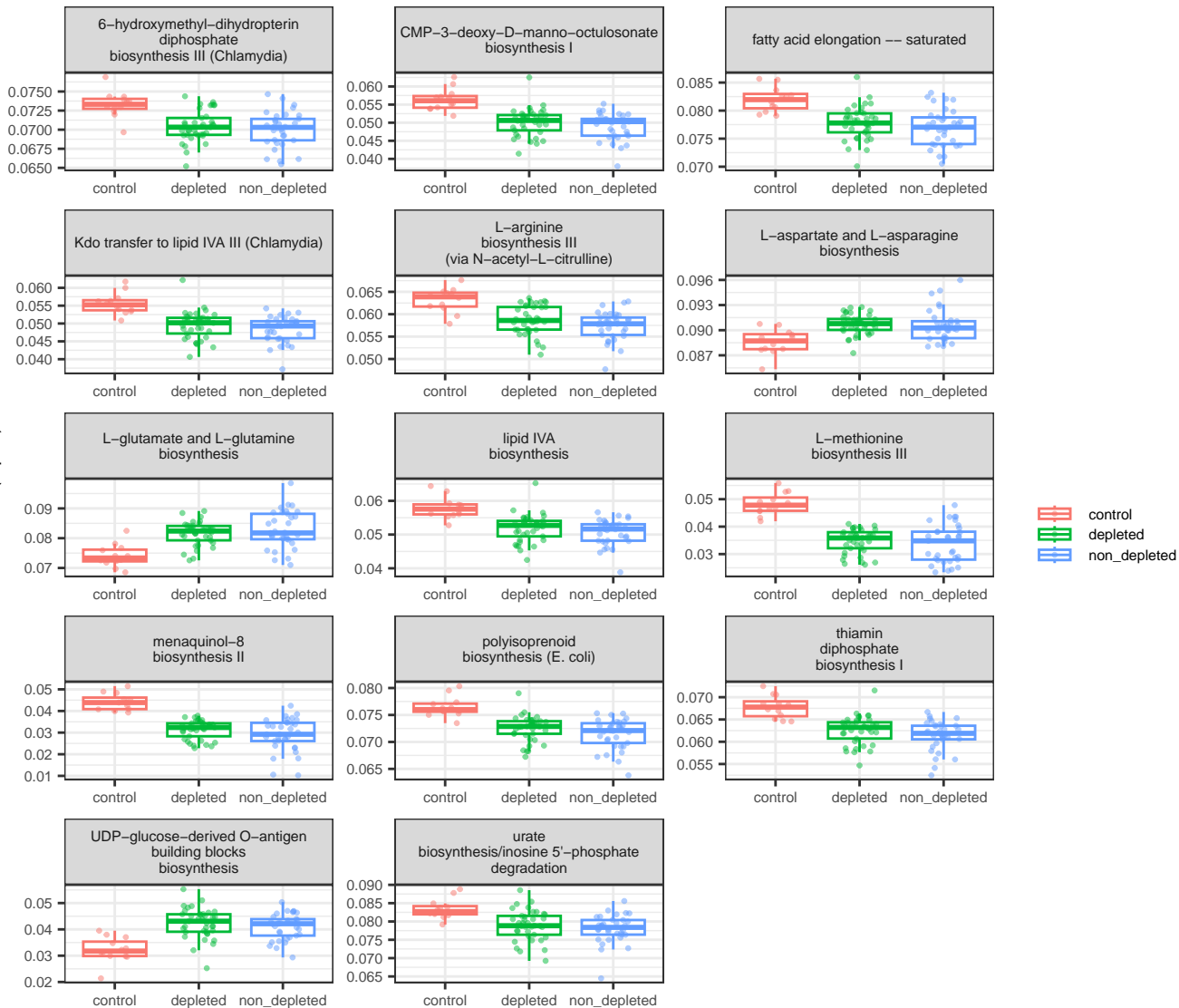

Supplement: SUPPLEMENTARY FIGURE S7 — [DFA_depl_nondepl_METAG] Effect of radiation dose and depletion treatment on the predicted relative abundances of metabolic pathways. Boxplots showing the variation of metabolic pathways whose relative abundance in the cecum of (A) the depleted group and (B) the non-depleted group differed between individuals exposed to low and high radiation dose. [file Data_Sheet_7.PDF]
