# Supplementary material for: Investigating the effects of radiation, T cell depletion, and bone marrow transplantation on murine gut microbiota
Source: Front Microbiol. 2024 Jun 5;15:1324403. doi: 10.3389/fmicb.2024.1324403 (PMC11188301; doi:10.3389/fmicb.2024.1324403)
Supplement: SUPPLEMENTARY FIGURE S6 — [DFA_all_METAG.pdf] Effect of radiation on predicted relative frequencies of phenotypic traits in caecum (CW) and ileum (IW) in controls and depleted or non-depleted irradiated mice. The differences between the three treatment groups were not statistically significant. [file Data_Sheet_6.PDF]

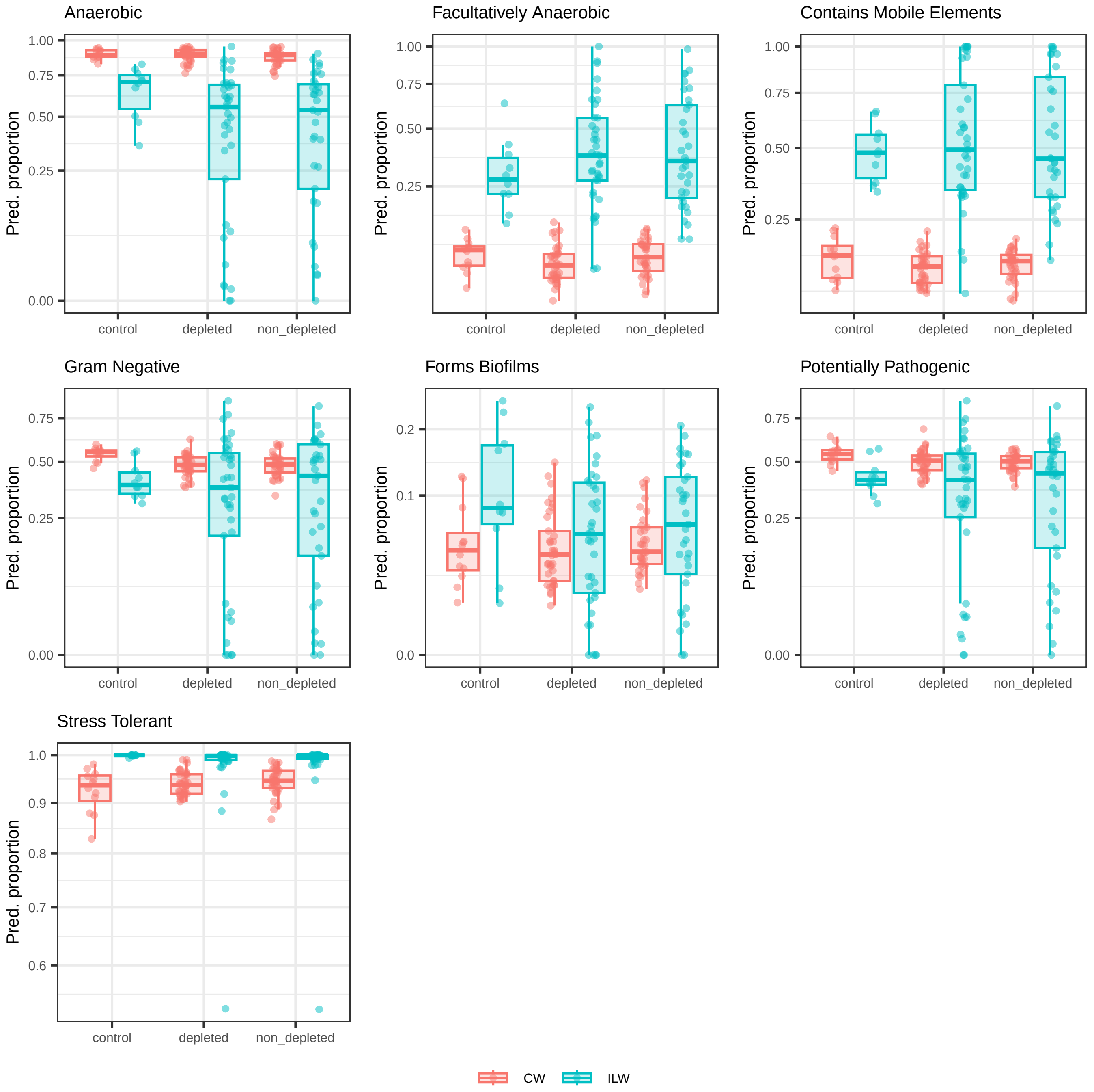
Anaerobic
Facultatively Anaerobic
Contains Mobile Elements
Gram Negative
Forms Biofilms
Potentially Pathogenic
Stress Tolerant
Pred. proportion
control
depleted
non_depleted
1.00
0.75
0.50
0.25
0.00
0.2
0.1
0.0
1.0
0.9
0.8
0.7
0.6
CW
ILW
